# CTGAN-Based Data Augmentation and XGBoost–LSTM Strength Prediction of CSG

**DOI:** 10.3390/ma19143150

**Published:** 2026-07-22

**Authors:** Guanghui Li, Yupeng Zhang, Qingqing Tian, Lei Guo, Qihui Chai

**Affiliations:** 1 School of Water Conservancy, North China University of Water Resources and Electric Power (Longzihu Campus), Zhengzhou 450046, China; kjyfb@hnsgyjy.com (G.L.); zhangyupeng689@163.com (Y.Z.); guoleiboss@126.com (L.G.); chaiqihui@ncwu.edu.cn (Q.C.); 2 Henan Water Conservancy Investment Group Co., Ltd., Zhengzhou 450002, China; 3 Henan Key Laboratory of Water Environment Simulation and Treatment, Zhengzhou 450002, China

**Keywords:** CTGAN, data augmentation, CSG, model performance, XGBoost–LSTM

## Abstract

**Highlights:**

- CTGAN can generate realistic and diverse CSG data, enhancing dataset diversity while preserving original features.
- The XGBoost–LSTM model predicts CSG strength with R^2^ of 0.9897, outperforming XGBoost and LSTM alone.
- Data augmentation improves model performance with better MAPE and RMSE metrics, validating effectiveness.
- This method reduces experimental work and lowers material consumption and engineering costs, improving efficiency.
- CTGAN data augmentation can be applied to other fields, addressing data scarcity and insufficient samples.

**Abstract:**

Cementitious sand and gravel (CSG) is commonly used in construction engineering; however, its mix proportion design is complex, and traditional physical experiments face limitations such as long cycles, high costs, and susceptibility to external factors when obtaining high-quality sample data. In this study, a foundational dataset was first acquired through physical experiments: 100 sets of CSG specimens with different mix proportions (cement content 40, 50, 60, 70 kg/m^3^; water-to-binder ratio 1.0, 1.2, 1.4; sand ratio 0.1, 0.2, 0.3, 0.4; fly ash content 20, 30, 40, 50 kg/m^3^) were prepared. After 28 days of standard curing, compressive strength and splitting tensile strength tests were conducted using a WAW-1000 electro-hydraulic servo universal testing machine, yielding 100 sets of real mechanical property data. The coefficients of variation for all test groups were below 10%, confirming the reliability and repeatability of the experimental data. On this basis, a data augmentation method based on Conditional Tabular Generative Adversarial Networks (CTGAN) is proposed. Through adversarial training between the generator and the discriminator, the model learns the multi-dimensional distribution characteristics of the original CSG data and generates 100 synthetic samples, which are then merged with the original data to expand the dataset to 200 samples. The quality of the synthetic data is evaluated using Wasserstein distance and correlation matrix heatmaps. Furthermore, a hybrid XGBoost–LSTM prediction model is proposed—XGBoost is used for feature construction to capture nonlinear interactions among mix proportion variables, and the constructed features are then fed into an LSTM network for sequential learning and regression prediction. The results show that the CTGAN-generated data are highly consistent with the original data in terms of kernel density distributions and variable correlations, with Wasserstein distance significantly superior to four comparative methods: Bootstrap, SMOTE, GaussianCopula, and TVAE. After augmentation, the XGBoost–LSTM model achieves a coefficient of determination (R^2^) of 0.9897 for compressive strength prediction (vs. 0.9793 before augmentation) and 0.9801 for splitting tensile strength (vs. 0.9882 before augmentation, a slight decrease). The mean absolute percentage errors (MAPE) are 4.49% and 4.11%, and the root mean square errors (RMSE) are 0.201 and 0.049, respectively; both error metrics are reduced compared with those before augmentation. Compared with baseline models including XGBoost, LSTM, Random Forest (RF), and Support Vector Regression (SVR), the XGBoost–LSTM model exhibits the best performance across all evaluation metrics, and Wilcoxon signed-rank tests confirm that the performance differences are statistically significant (*p* < 0.05). The proposed method of CTGAN-based data augmentation combined with the XGBoost-LSTM hybrid model provides an effective solution to the problem of insufficient CSG sample data and offers a reference for data enhancement and performance prediction of other small-sample materials.

## 1. Introduction

Cementitious sand and gravel (CSG) is a new type of dam construction material that is economical, safe, environmentally friendly, and low-carbon [1,2]. It is obtained by mixing cementitious materials [3], water, riverbed sand and gravel, or excavation waste materials using simple equipment [4,5]. Compressive strength and tensile strength are important indicators of CSG, and researchers currently mainly conduct related studies using experimental methods. However, the experimental process is time-consuming, costly, and susceptible to external factors. Moreover, due to the complex composition and heterogeneity of CSG, obtaining a sufficient amount of high-quality CSG sample data has always been a challenge. The lack of sufficient data can result in inaccuracies in modeling and prediction.

With the continuous advancement of technology, data augmentation methods play an increasingly important role in addressing data scarcity. For example, Kim et al. used StyleGAN to resolve imbalanced manufacturing data [6], while Raviv and Shlezinger and Bayer et al. applied augmentation techniques to deep receivers and text classification, respectively [7,8]. Similarly, Jeon et al. and Mazzini et al. demonstrated the effectiveness of data augmentation in wearable sensor data distillation and pavement distress segmentation [9,10]. In recent years, traditional Generative Adversarial Networks (GANs) have seen extensive development. Comprehensive reviews by Saxena and Cao and Alqahtani et al. detail their broad applications and existing challenges [11,12]. Fundamental advancements have also been made in game-theoretical modeling [13] and network architecture optimization [14]. Furthermore, GANs have achieved great success in complex computational tasks, such as evolutionary multi-objective optimization [15] and foggy image semantic segmentation [16]. However, despite these successes in image and algorithmic domains, they have encountered challenges in dealing with structured tabular data. However, they have encountered challenges in dealing with structured tabular data. Tabular data consists of columns with different types, conditional constraints, and complex correlations, making it crucial to create a model that accurately captures these characteristics. CTGAN is a neural network architecture composed of a generator and a discriminator. By introducing a conditional generator and employing an adversarial training strategy, the model can generate realistic synthetic data based on specified conditional information [17], a technique successfully applied to generating electronic health records and clinical trial data [18,19]. Leveraging this mechanism, CTGAN enables the efficient generation of tabular data with specific attributes. This provides crucial data support for building robust intrusion detection systems in smart IoT environments [20,21], thereby widely meeting diverse practical application requirements [22]. Additionally, CTGAN leverages adversarial training between the discriminator and the generator to improve the quality and diversity of the synthetic data. CTGAN has been widely applied in various fields, including data privacy protection [23], data augmentation [24], data analysis [25,26], and model evaluation [27]. Its emergence provides researchers and data scientists with a powerful tool to generate synthetic tabular data with authenticity and diversity.

Machine learning is currently in a golden age and is rapidly developing as one of the core components of artificial intelligence. The XGBoost algorithm, as one of the three main algorithms in machine learning, is efficient and fast, and is widely used in various fields such as finance [28,29,30], hydrology [31,32,33], materials [34,35], automation [36,37,38], and data mining. It has demonstrated good learning performance and accuracy. Several studies have applied machine learning to CSG strength prediction. For instance, researchers have used the XGBoost model to select variables with high correlation to the splitting tensile strength of CSG as input features, and combined the predicted results with the original features in a Random Forest (RF) model, demonstrating the effective prediction of CSG splitting tensile strength [39]. Additionally, Long Short-Term Memory (LSTM) is a special type of Recurrent Neural Network (RNN) that can learn long-term dependencies and solve the problems of vanishing and exploding gradients in traditional neural networks [40]. In recent years, LSTM has been extensively developed and successfully applied across diverse fields, including optical transmission systems [41], hydrological time series forecasting [42], ship speed prediction [43], concrete damage assessment [44], and anomaly detection in the industrial IoT [45]. Furthermore, it has been combined with Graph Neural Networks (GNN) to form GNN-LSTM composite models, which have been applied to water quality prediction and achieved significant performance improvements [46,47]. Machine learning is also applied in strength prediction of civil engineering materials. For example, XGBoost has been used to predict the compressive strength of concrete, achieving high accuracy, but excessive hidden layers may lead to gradient vanishing [48]; LSTM has been used for concrete compressive strength prediction, demonstrating its superiority and avoiding the gradient vanishing problem [49]. While these studies demonstrate the feasibility of machine learning for CSG and civil engineering materials, they rely on relatively small experimental datasets without addressing the fundamental challenge of data scarcity. Additionally, single-model approaches such as XGBoost may suffer from gradient vanishing when network depth increases, while LSTM models, though capable of capturing long-term dependencies, may not fully exploit feature interactions in tabular CSG data. To our knowledge, no prior study has applied Generative Adversarial Network-based data augmentation to address the data scarcity problem specific to CSG materials. Therefore, a critical scientific gap exists: there is no established method for augmenting limited CSG experimental datasets to improve the accuracy and robustness of machine learning-based strength-prediction models.

Based on the background mentioned above, this study proposes a CSG data augmentation method based on Conditional Tabular Generative Adversarial Networks (CTGAN) with the aim of expanding and enhancing the dataset by generating synthetic CSG samples. Enhancing CSG, a complex material, poses challenges because its physical properties and structure need to be accurately preserved during the generation process. In our method, we first collect a set of real CSG sample data as the training set. Then, we design a generator and a discriminator and iterate the training process, allowing the generator to gradually learn the distribution of real sample data and generate synthetic samples with similar features. To evaluate the effectiveness of our proposed data augmentation method on CSG sample classification tasks, we propose a composite model, XGBoost–LSTM, to predict the compressive strength and splitting tensile strength of CSG before and after augmentation. We compare the results with those of the baseline models, XGBoost and LSTM, as well as with the results of the XGBoost model on the original dataset. The novelty and scientific contributions of this study are threefold: (1) This study is the first to apply CTGAN to CSG data augmentation. Unlike conventional methods (e.g., SMOTE, Bootstrap) that rely on linear interpolation or resampling, CTGAN leverages mode-specific normalization and conditional generation to simultaneously capture the multimodal distributions and nonlinear inter-variable correlations inherent in heterogeneous composite material data. This represents a methodological advance for addressing data scarcity in experimental materials science, where each data point requires costly and time-consuming physical testing. Furthermore, the decoupling verification experiment designed in this study—which isolates the contribution of data quality from data quantity—provides a rigorous and transferable evaluation paradigm for validating generative augmentation methods; (2) A composite XGBoost–LSTM model is proposed that introduces a two-stage learning paradigm for CSG strength prediction: the first stage employs XGBoost to construct enriched higher-order feature representations by capturing the nonlinear synergistic and antagonistic interactions among mixture components (e.g., cement–water–binder ratio interactions governing hydration, fly ash–cement ratio influencing pozzolanic reaction kinetics), and the second stage feeds these constructed features into LSTM for hierarchical feature refinement through its gating mechanisms. To our knowledge, no prior study has combined gradient boosting-based feature construction with recurrent network-based sequential feature refinement for CSG strength prediction; (3) The proposed framework—combining CTGAN-based data augmentation with hybrid machine learning prediction—is generalizable to other domains where experimental data are scarce and characterized by complex multivariate dependencies, such as ultra-high performance concrete, fiber-reinforced polymers, and geopolymer composites. The comprehensive evaluation methodology incorporating Wasserstein distance, correlation matrix analysis, and rigorous statistical testing sets a transferable standard for validating data augmentation quality in materials science.

## 2. Materials and Methods

### 2.1. Raw Materials and Mix Ratio

**Figure 1:**
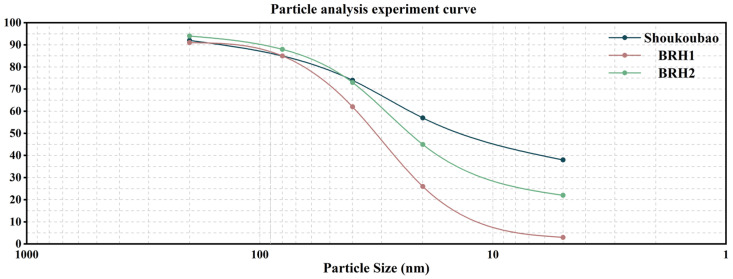
Aggregate particle size distribution curve.

The main materials used in this experiment include natural gravel, cement, fly ash, natural river sand, and water. The coarse aggregate is natural river gravel, collected from the Beiru River riverbed in Pingdingshan City, Henan Province. The gravel has a two-grade particle size distribution with a medium-to-fine ratio of 3:2, where the medium-sized particles range from 20 mm to 40 mm and the fine-sized particles range from 5 mm to 20 mm. The gravel exhibits a crush value of 2.74%, a water content of 0.43%, a water absorption of 0.68%, a bulk density of 1669.3 kg·m^−3^, and an apparent density of 2350 kg·m^−3^, as shown in Table 1. The particle size distribution curve of the aggregate is shown in Figure 1. Among them, BRH1 is the bottom curve, representing the washed material; BRH2 is the middle curve, representing the raw material; and the topmost grading curve represents the raw material for the Shoukoubao project. The fine aggregate is sand purchased from Xinmiao Sandstone Co., Ltd. (Nanyang, China) in Tanghe County, with a fineness modulus of 2.94, classified as medium sand. The particle size distribution table of the sand is shown in Table 2. The fly ash used in the experiment was dry-disposed Class II grade F-type fly ash, and its performance is shown in Table 3. The cement used is P·O42.5-grade cement from Tianrui Cement Group Co., Ltd. (Ruzhou, China), and the fly ash was purchased from Yulian Power Plant. Its physical and mechanical properties are shown in Table 4.

This experiment refers to the “Study on Super-Poor Cemented Materials Dam [50]”. The cement dosage is set at 70, 60, 50, and 40 kg/m^3^, and the “excess replacement method” is used when adding fly ash. According to the calculations based on the excess replacement method, the fly ash dosages used in this experiment are 20, 30, 40, and 50 kg/m^3^, respectively. Referring to the Shoukoupo Project in Shanxi Province (which adopted a water-to-binder ratio of 1.58), the water-to-binder ratios in this experiment are set at 1.0, 1.2, and 1.4. The gravel-to-sand ratio affects the compactness of the specimens and the cementing properties of the materials. In this experiment, gravel-to-sand ratios of 0.1, 0.2, 0.3, and 0.4 are selected. According to the “Hydraulic Concrete Test Procedures” (SL/T 352-2020) [51], the proportion of gravel to small stones is set at 6:4. The specific size range of the small stones is 5 to 20 mm. A total of 100 sets of proportions for cemented gravel are designed, as shown in Table 5.

### 2.2. Specimen Design and Preparation

**Figure 2:**
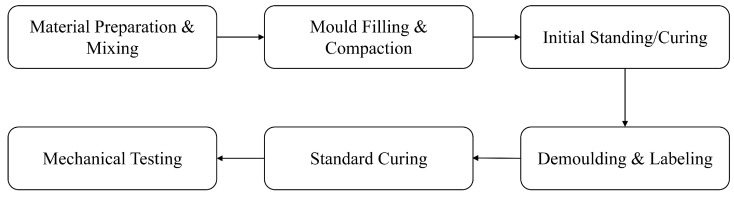
Experimental workflow of cemented sand and gravel (CSG) specimen preparation.

This experiment refers to the “Technical Guidelines for Cemented Particle Dam Construction” (SL/T 678-2025) [52], “Hydraulic Concrete Test Procedures” (SL/T 352-2020), and “Standard Test Methods for Long-Term Performance and Durability of Ordinary Concrete” (GB/T 50082-2024) [53]. The specimen size is a standard cube of 150 mm × 150 mm × 150 mm, and the manufacturing process is shown in Figure 2.

(1) Material Preparation and Mixing: Weigh the materials according to the calculated quantities. Before mixing, add the measured cementitious materials and aggregates to the mixer according to the experimental mix proportion. Dry-mix for 10 s to ensure thorough and uniform blending, then add the accurately measured water. Cover the drum of the mixer and continue mixing for 2 min.
(2) Mold Filling and Compaction: Before filling the molds, evenly coat the inner walls with machine oil. Then, divide the well-mixed CSG material into two layers and fill the pre-oiled molds, ensuring approximately equal thickness for each layer. During filling, manually tamp the material at least 25 times per layer. The specimens are compacted and vibrated twice during the filling process. In this experiment, roller compaction is applied using a magnetic vibration table to secure the iron mold, preventing lateral movement and ensuring uniform amplitude during vibration.
(3) Initial Standing/Curing: After forming, place the specimens in a room at 20 °C ± 5 °C and let them stand for 24 to 48 h for initial curing.
(4) De-molding and Labeling: After the standing period, remove the molds and label each specimen for identification.
(5) Standard Curing: Immediately transfer the labeled specimens to a curing room with controlled temperature (20 °C ± 2 °C) and relative humidity above 95%. Cure them there until the age of 28 days.
(6) Mechanical Testing: At 28 days, conduct compressive and splitting tensile strength tests using the WAW-1000 electro-hydraulic servo universal testing machine (Shanghai Huarong Test Instrument Co., Ltd., Shanghai, China). The testing procedures follow the standard methods described in Section 2.2.

**Figure 3:**
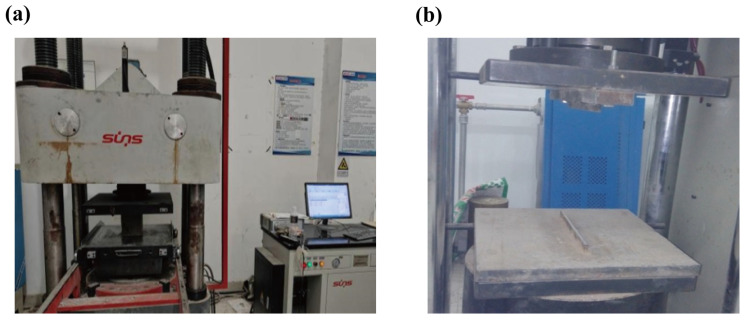
Compression and splitting test equipment ((**a**) compression strength testing equipment and (**b**) splitting strength testing equipment).

The pressure-testing machine used in the compressive strength test is the WAW-1000 electro-hydraulic servo universal testing machine, as shown in Figure 3a. The test process is automatically controlled by a computer, and the compressive strength test is conducted on the cemented gravel specimens. After the specimens reach the specified age in the curing room and are removed and wiped clean, they are verified to be error-free. Then, they are accurately placed at the center of the lower pressing plate of the testing equipment to ensure that the pressure-bearing surface is perpendicular to the top surface during the molding process. During the test, continuous and uniform pressure is applied. When the sample is approaching failure, it undergoes accelerated deformation until it completely fails. During this period, the failure load of the sample is recorded in detail.

The splitting test is carried out by applying a uniformly distributed load on the surface of the specimen using specially made steel pads, in order to achieve the purpose of splitting the specimen. To conduct the splitting tensile test, the testing machine needs to be modified accordingly, so that the surface load of the testing machine is transformed into a uniformly distributed linear load, and during the transformation process, the pad itself should not deform to avoid uneven loads during the load transformation process, which may affect the accuracy of the test results. The modification of the pressure-testing machine is shown in Figure 3b. Before the test, the specimen should be wiped clean, the appearance of the specimen should be inspected, the position of the splitting surface of the specimen should be accurately determined and the size of the splitting surface should be measured, and the specimen should be placed in the center position of the pressing plate of the pressure-testing machine. Place the pad in-between the upper and lower pressing plates and the specimen, with the direction of the pad being perpendicular to the top surface when the specimen was formed. Align the lower pad with the lower end points of the two parallel reference lines. Operate the pressure machine to make the upper pressing plate descend at a loading rate of 0.01 MPa/s and load continuously and uniformly until the specimen breaks. Click “Exit Loading” to stop the test and record the peak load.

During the experiment, in order to ensure the accuracy and reliability of the test data, the representative values of the performance tests for cemented gravel were determined. When the difference between the single measurement value and the intermediate value is within 20% of the intermediate value, the average value of the test results of 3 specimens is taken as the test result of the compressive strength of this group of specimens. If the difference between one measurement value and the intermediate value exceeds 20% of the intermediate value, the intermediate value is taken as the test result. If the difference between two measurement values and the intermediate value exceeds 20% of the intermediate value, the test result of this group is invalid. In order to ensure the reliability and stability of the test results, each mix ratio was tested three times in repetition. The arithmetic mean of the test results was taken as the final representative value. Furthermore, the coefficient of variation was calculated for the results of different test groups. The results showed that the coefficient of variation of the strength data under different mix ratios was always lower than 10%, indicating that the test results had good repeatability.

### 2.3. Data Normalization

In the data normalization step before data augmentation, clustering analysis and probability density estimation are first conducted on each column of the CSG mix proportion and strength data. Assuming that each column of data follows a Gaussian distribution, the number of estimated modes is used to reveal the distribution pattern of the data. This can be achieved by employing a Variational Gaussian Mixture Model to learn the data distribution, using parameters $\mu_{k}$ and $\varphi_{k}$ to represent the weights and standard deviations of the k-th mode. For each value $b_{i,j}$ in each column of data *B_i_*, the probability density ρk under each mode is calculated as shown in Formulas (1) and (2).

(1) $$B_{i}(c_{i,j})=\sum_{k=1}^{m_{i}}\mu_{k}N\left(c_{i,j},\eta_{k},\phi_{k}\right)$$

(2) $$\rho_{k}=\mu_{k}N\left(c_{i,j},\eta_{k},\phi_{k}\right)$$

where *m_i_* is the number of patterns, and $\mu_{k}$ and $\varphi_{k}$ are the weight and standard deviation of the *k*-th mode.

By computing the probability density under each mode, we can determine the probabilities of each value belonging to different modes. Identify the mode ρk with the highest probability density in mode mi and normalize it.

For example, if among the three modes $\eta_{1},\;\;\eta_{2},\eta_{3}$ in *m_i_*, $\eta_{2}$ has the highest probability density, then the values *c_i_*_,_*_j_* can be represented as the unique thermal vector [0, 1, 0] and the value of the mode can be represented by the scalar $\alpha_{i,j}=\left(c_{i,j}-\eta_{2}\right)/\varphi_{2}$. This converts the pre-enhancement data into a standardized form centered on the highest probability density pattern. Each column is concatenated to represent the entire table data. The *r_j_* representation of each row is obtained by concatenating the normalized representation of each column. Concatenation operations can be expressed using the symbol ⨁, as in Formula (3):

(3) $$r_{j}=\alpha_{1,j}⊕\beta_{1,j}⊕\ldots ⊕\alpha_{Nc,j}⊕\beta_{Nc}$$

where *r_j_* represents the representation of the *j*-th row, and ⨁ denotes the concatenation symbol.

Through the above steps, the data is transformed into a normalized representation centered around the mode with the highest probability density and represented by concatenating the representations for each column, which represents the entire table data. This data normalization method helps highlight the patterns and regularities in the data, providing a more accurate and comparable data representation for subsequent data augmentation and model training.

### 2.4. Data Preprocessing

In the process of data preprocessing, the min–max normalization method is used to normalize the numerical features of CSG data before data augmentation. The expression is:

(4) $$y_{i}=\frac{x_{i}-x_{\min}}{x_{\max}-x_{\min}}$$

where *y_i_* is the normalized data, *x*_min_ is the minimum data in the sample; *x*_max_ represents the maximum feature data; and *x* represents data before normalization.

During normalization, no features are discarded or deleted; all numerical features are normalized. This ensures data integrity and all features can be used in subsequent data enhancement and model training. Through min–max standardization, parameter values are converted to values in the range [0, 1]. This maps the data to a unified range, enabling comparability between different features. The model can understand the relationships between features more accurately, thus improving its accuracy and stability. After normalization, the data is transformed from a dimensioned expression to a dimensionless one. This means that the numerical value of the feature is no longer dependent on the specific original unit of measure but is represented within a uniform range. Such expression is conducive to the comparison and synthesis of features and further enhances the effect of the model. Through the above data preprocessing steps, CSG data becomes numerically comparable while retaining the integrity of all features. This lays the foundation for subsequent data enhancement and modeling processes, contributing to the accuracy and stability of the model.

### 2.5. Methodology

#### 2.5.1. CTGAN

GAN and CTGAN are generative models based on the theory of zero-sum games. GAN consists of a generator *G* and a discriminator *D* [54]. During the training process, the generator continuously improves its ability to generate fake data to deceive the discriminator, while the discriminator determines whether the input data is real or generated. These two components iteratively optimize each other until a dynamic equilibrium is reached. The generator ultimately generates simulated samples and completes data enhancement. The loss function of GAN is shown in Equation (5):

(5) $$\underset{G}{\min}\;\underset{D}{\max}V\left(G,D\right)=E_{X\sim P_{r}}\left\{\log{\left\{\left[D\left(x\right)\right]\right\}}_{Z\sim P_{Z}}\log\left\{\left[1-D\left(G\left(z\right)\right)\right]\right\}\right\}$$

where *x* represents real sampling, *P_r_* represents real sampling distribution, z represents random noise, *P_z_* represents random noise distribution, *G*(z) represents false sample data generated by generator *G*, and *D*(·) represents output value of discriminator *D*.

**Figure 4:**
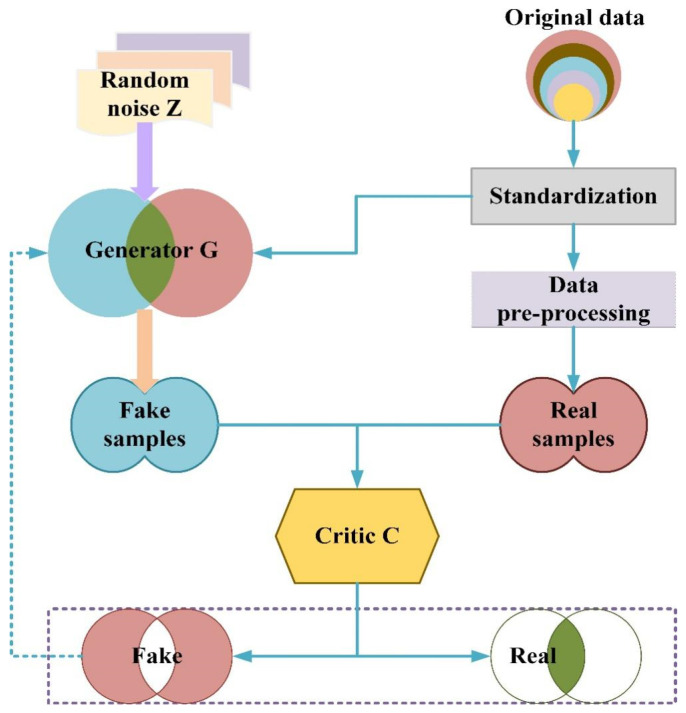
CTGAN architecture for CSG tabular data augmentation.

Although GAN can generate synthetic samples that conform to the distribution of real data, it is not suitable for generating tabular data. CTGAN is a generative model based on GAN that has been optimized for the generation of tabular data [55]. CTGAN considers the conditional information in tabular data and employs specialized generator and critic structures, along with other techniques. The architecture of CTGAN is illustrated in Figure 4. CTGAN consists of two neural networks: the generator G and the critic C (similar to the discriminator in the classical GAN structure). To overcome the non-Gaussian and multimodal distributions in continuous columns of tabular data, CTGAN adopts mode-specific normalization. It addresses the issue of imbalanced categories in discrete columns through conditional generation and sample training. Additionally, CTGAN incorporates some recent advancements in GAN training, such as the loss function of WGAN-GP [56] and the critic structure of PacGAN [57], to improve training stability and the quality of generated data. The loss function of CTGAN is shown in Equation (6) [58]:

(6) $$L=E_{G\left(z\right)\sim P_{g}}\left[D\left(G\left(z\right)\right)\right]-E_{X\sim P_{r}}\left[D\left(x\right)\right]+\lambda E_{y\sim P_{y}}\left[{\left(\left\|\nabla_{y}\left(D_{y}\right)\right\|-1\right)}^{2}\right]$$

where *y* represents the sample for linear interpolation of the real data *x*, λ represents the gradient penalty factor, and *P_r_* and *P_g_* represent the distribution of the real and generated data, respectively.

A CSG dataset is tabular data that contains both numerical and categorical information. CTGAN is specifically designed for generating tabular data and can effectively learn the distribution of CSG data to generate synthetic samples that match the distribution of real data. This is useful for data augmentation while maintaining data availability.

#### 2.5.2. Training Process

First, prepare the original CSG dataset as training data. Ensure that the dataset includes features relevant to CSG attributes. Then, install the necessary dependencies and import them. Preprocess the data before augmentation, ensuring that it is ready as input for CTGAN. Configure the input parameters of the CTGAN model based on the features of the CSG dataset, such as feature dimension and the settings of the generator and critic (discriminator) hidden layers. Train the CTGAN model using the prepared data. During the training process, CTGAN will learn the distribution characteristics of the original data and generate a synthetic dataset. Use the trained CTGAN model to generate additional synthetic data. You can specify the desired increase in data quantity by calling the generation function in the generator.

Based on the Conditional Tabular Generative Adversarial Networks technology, data is enhanced using the pre-augmentation dataset. The generator produces data. First, train the generator and discriminator using the pre-augmentation dataset. The generator generates fake data, the discriminator evaluates the authenticity of the data, and provides feedback to the generator to improve the generation quality. This training process adopts the adversarial training method, i.e., achieving balance by minimizing the loss function between the generator and the discriminator. Then, generate enhanced data. After training is complete, the generator can be used to generate enhanced data with a realistic table structure and content. Sample m samples from the real samples {*x*_1_, *x*_2_, … $x_{m}$}, sample m noise samples from the prior distribution {*z*_1_, *z*_2_, … $z_{m}$} and obtain m generated samples through the generator {*x*_1_, *x*_2_, … $x_{m}$}, and use the generator to generate tabular data.

The data generated by the generator is fed into the discriminator for a discrimination score. The discriminator checks whether the generated data has a score of 0.5; if not, the discriminator provides feedback to the generator. The discriminator is based on the cross-entropy formula:

(7) $$H{\left(\left(x_{i},y_{i}\right)\right.}_{\left(i=1\right)}^{N},\left.D\right)=-\sum_{i=1}^{N}\left(1-y_{i}\right)\log\left(1-D\left(x_{i}\right)\right)$$

If we assume a correct sample distribution, then the corresponding (1 − *y_i_*) is the distribution of the generated sample. *D* represents the discriminator, then *D*(*x_i_*) represents the probability that the discriminator sample is correct, and (1 − *D*(*x_i_*)) corresponds to the probability that the discriminator sample is wrong. When the discriminant probability is 0.5, the generated data can be output.

If yes, output the generated data; otherwise, the discriminator feeds it back to the generator, and the above steps are repeated until the termination criteria are met, i.e., until the generated data has a discrimination score of 0.5, and then the final generated data is output. The new samples generated by the tabular adversarial network are combined with the pre-augmentation dataset to form an augmented dataset with a larger data scale. Ensure that the combined data conforms to the same distribution characteristics as the pre-augmentation data, thus maintaining the representativeness of the data.

#### 2.5.3. CSG Intensity Prediction Model

According to previous studies, the XGBoost algorithm can be used to predict CSG splitting tensile strength [59]. The XGBoost algorithm is a strong classifier based on the GBDT algorithm and Boosting, which consists of several weak classifiers. Its ensemble model is shown in Equation (8):

(8) $${\hat{y}}_{i}^{\left(t\right)}=\sum_{k=1}^{K}f_{k}\left(x_{i}\right),f_{k}\in F$$

where ${\overset{\wedge }{y}}_{i}^{\left(t\right)}$ is the model prediction in the *t*-th round, *k* is the number of trees contained in the model, *f_k_* is the correlation between the structure of the *k*-th tree and the leaf weights (ω), $x_{i}$ is the feature represented by the *i*-th tree in the model, and *F* is the space where the model contains the tree.

LSTM uses memory cells and gates to control long-term information and can extract long- and short-term correlations in time series, effectively obtaining data features. The main structure of LSTM includes the forget gate, input gate, update gate, and output gate. The main formulas of its structure are as follows:

(9) $$f_{t}=\sigma \left(W_{f}\left(h_{t-1},x_{t}\right)+b_{f}\right)$$

(10) $$i_{t}=\sigma \left(W_{i}\right)\left(h_{t-1},x_{t}+b_{i}\right)$$

(11) $$g_{t}=\tanh\left(W_{g}\left(h_{t-1},x_{t}\right)+b_{g}\right)$$

(12) $$c_{t}=f_{t}c_{t-1}+i_{t}g_{t}$$

(13) $$o_{t}=\sigma \left(W_{o}\left(h_{t-1},x_{t}\right)+b_{0}\right)$$

(14) $$h_{t}=o_{t}\tanh\left(c_{t}\right)$$

where *f_t_*, *i_t_*, *g_t_* and *o_t_* respectively represent the output values of the forgetting gate, the input gate, the update gate and the output gate at the current moment; *W_f_*, *W_i_*, *W_g_* and *W_o_* represent weight vectors; and *b_f_*, *b_i_*, *b_g_* and *b_o_* represent the deviation vector. *h_t_* indicates the hiding condition at the current time. *h_t_*_−1_ indicates the hiding condition of the previous moment. *c* represents the cell state at the current moment; *c_t_*_−1_ indicates the cell status at the previous time. *x_t_* represents the input at the current time; σ stands for Sigmoid activation function; tanh represents the original neural network layer.

**Figure 5:**
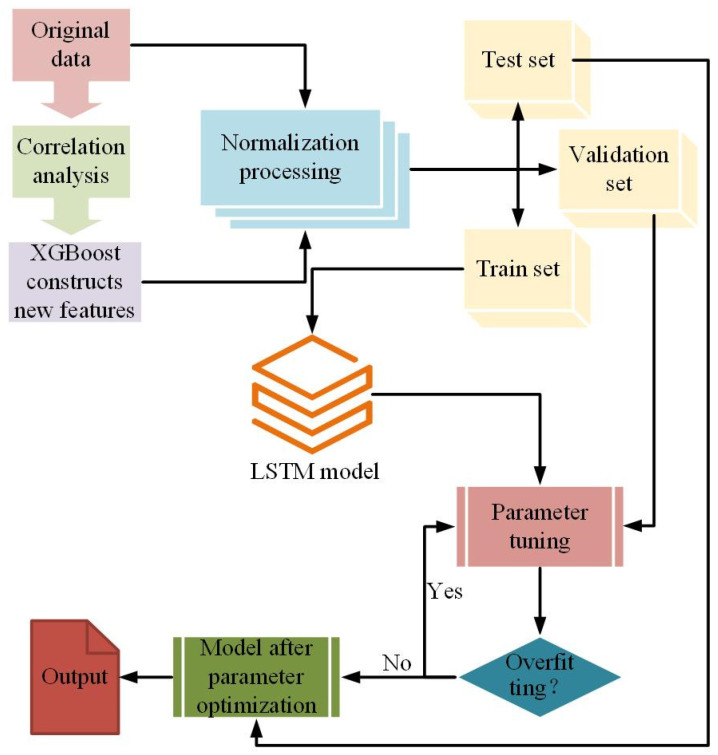
XGBoost–LSTM hybrid prediction framework for CSG strength estimation: feature construction by XGBoost followed by sequential modeling using LSTM.

As shown in Figure 5, the prediction process of the XGBoost–LSTM model begins with a correlation analysis of the original data to identify the relationships between data points. Subsequently, the XGBoost model is utilized to construct new features, enhancing the model’s predictive capabilities. After feature construction, all features undergo normalization to ensure uniformity and consistency in the model’s inputs. The CSG sample data are randomly divided into training, testing, and validation sets in a ratio of 7:2:1. The processed training data are then fed into the LSTM model, a neural network particularly well-suited for handling sequential data. To optimize the model’s performance, hyperparameters of the LSTM model are fine-tuned using a grid search method. This process involves randomly combining parameters within a given range and performing cross-validation on the validation set to evaluate the model’s effectiveness for different parameter combinations and to determine the optimal parameter set. This step is crucial for preventing overfitting and improving the model’s generalization ability.

Under the optimal parameter configuration, the XGBoost–LSTM model achieves a coefficient of determination (R^2^) of 0.9897, indicating that the model can predict the compressive strength of CSG efficiently and accurately. This high-performance metric proves the model’s effectiveness and reliability in predicting the compressive strength of CSG. Finally, the model is evaluated on the test set to verify its predictive performance, ensuring the model’s accuracy and stability in practical applications [60].

## 3. Results

### 3.1. Summary of Laboratory Experimental Results

**Figure 6:**
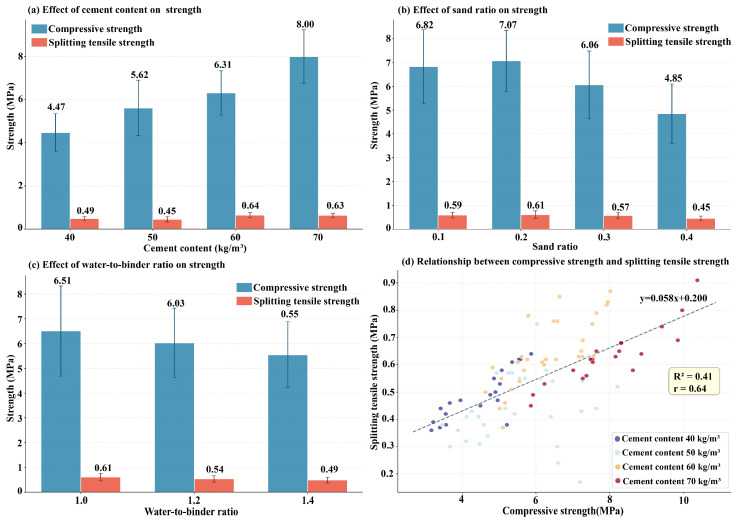
Laboratory experimental results of CSG specimens: (**a**) effect of cement content on com-pressive and splitting tensile strength; (**b**) effect of sand ratio on strength; (**c**) effect of water–binder ratio on strength; (**d**) correlation between compressive strength and splitting tensile strength.

Figure 6 presents the key laboratory experimental results of the 100 sets of CSG specimens. As shown in Figure 6a, both compressive and splitting tensile strength increase consistently with increasing cement content; the mean compressive strength rises from 4.28 MPa at 40 kg/m^3^ to 7.87 MPa at 70 kg/m^3^, and the mean splitting tensile strength increases from 0.46 MPa to 0.63 MPa over the same range, confirming that cement content is the dominant factor governing CSG strength development. Figure 6b indicates that a sand ratio of 0.2 yields the highest strength values, with a mean compressive strength of 6.80 MPa and a mean splitting tensile strength of 0.64 MPa; beyond this optimal ratio, both strength indicators decline as sand ratio increases to 0.4, suggesting that excessive sand content reduces the compactness and cementing properties of the specimens. As shown in Figure 6c, both compressive and splitting tensile strength decrease with increasing water–binder ratio; at a water–binder ratio of 1.0, the mean compressive strength reaches 6.54 MPa and the mean splitting tensile strength reaches 0.58 MPa, while these values progressively decline at water–binder ratios of 1.2 and 1.4, consistent with the expected dilution effect on the cementitious paste. Figure 6d presents the correlation between compressive and splitting tensile strength, with data points color-coded by cement content level. A linear regression yields y = 0.058x + 0.200 with R^2^ = 0.41 and a Pearson correlation coefficient r = 0.64, indicating a moderate positive correlation. Notably, data points corresponding to higher cement contents (green region) cluster in the upper-right area of the plot, exhibiting both higher compressive and tensile strength values, while lower cement content groups (blue region) concentrate in the lower-left area, further corroborating the dominant influence of cement content on CSG mechanical performance.

### 3.2. Experimental Setup

#### 3.2.1. Data Augmentation Methods

To comprehensively evaluate the data augmentation effect of CT-GAN, this paper selects five comparison methods: Bootstrap (with 100 resampling groups with replacement as the baseline for sample size increase), SMOTE (standardized and based on five-nearest neighbor linear interpolation to generate 100 groups), GaussianCopula (using the SDV library, Gaussian Copula modeling), TVAE (using the SDV library, variational autoencoder), and CT-GAN (using the SDV library, conditional Generative Adversarial Network). Each method generates 100 sets of data, which are consistent with the original data size. The parameter configurations are shown in Table 6.

#### 3.2.2. Prediction Models

Three prediction models, namely XGBoost, LSTM and XGBoost–LSTM, were adopted for performance evaluation. For XGBoost, the number of decision trees was set to 100, the learning rate was 0.05, the maximum tree depth was six, and the subsample ratio was 0.8. The LSTM network contained 64 LSTM units and a 32-dimensional fully connected layer with a Dropout rate of 0.2. Adam optimizer and MSE loss function were used. The model was trained for 100 epochs with a batch size of 16 and an early stopping patience of 20. For the XGBoost–LSTM hybrid model, the prediction results of XGBoost were constructed as new features and concatenated with original features, then imported into the LSTM network with the same structure as the single LSTM model. This hybrid model combined the advantages of ensemble learning and deep learning to capture complex feature interactions. The detailed parameter settings are listed in Table 7.

#### 3.2.3. Experimental Design and Evaluation Metrics

Three groups of experiments were designed to systematically verify the effectiveness of the CT-GAN-based data augmentation method:

(1) Distribution quality evaluation: Wasserstein distance and correlation matrix heatmaps were adopted to compare the statistical distribution differences between original data and data generated by different augmentation methods.

(2) Decoupling verification of sample size and data quality: The original 100 groups of data were fixed as the training set, and 100 groups of data generated by each augmentation method were used as independent test sets. The XGBoost–LSTM model was adopted for five repeated training runs with different model initializations. The mean value and standard deviation of the coefficient of determination R^2^, as well as the 95% confidence interval, were calculated. Paired *t*-test was used to analyze the performance differences between CT-GAN and other methods.

(3) Comprehensive performance comparison after data augmentation: The original 100 groups of data were combined with 100 groups of synthesized data generated by each method respectively to form six datasets with 200 samples in total. A 10-fold cross-validation was conducted using the XGBoost–LSTM model. Four evaluation indicators including R^2^, RMSE, MAE and MAPE were adopted. The mean value, standard deviation and 95% confidence interval (calculated via 1000 Bootstrap re-samplings) were computed. Wilcoxon signed-rank test was applied to compare the R^2^ differences between CT-GAN and the second-best method.

### 3.3. Evaluation of Generated Data Distribution Quality

**Figure 7:**
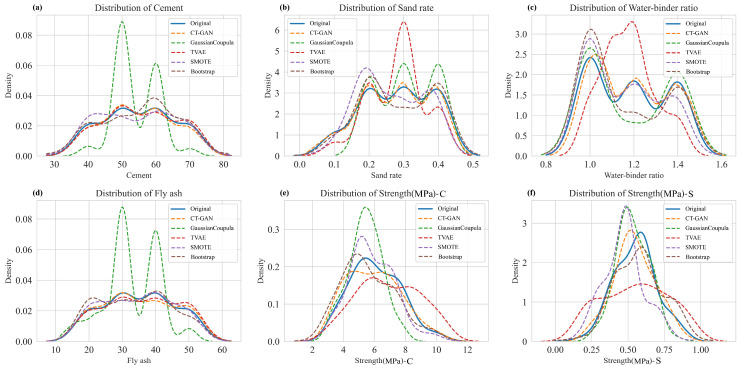
Kernel density distribution comparisons of generated data across different augmentation methods ((**a**) cement content; (**b**) sand ratio; (**c**) water–binder ratio; (**d**) fly ash content; (**e**) compressive strength; (**f**) splitting tensile strength).

To quantitatively assess the statistical fidelity of data generated by CT-GAN, Wasserstein distance and correlation matrix heatmaps were utilized to compare the distribution characteristics of original data and data produced by Bootstrap, SMOTE, TVAE, GaussianCopula and CT-GAN. Bootstrap only resampled original data with replacement without generating new information. SMOTE created new samples through linear interpolation in the feature space. TVAE and GaussianCopula are deep learning-based tabular data generation models. Figure 7 presents the average Wasserstein distance and kernel density distribution of six continuous features, including cement content, sand ratio, water–binder ratio, fly ash content, compressive strength and splitting tensile strength.

It can be seen from the kernel density curves of six continuous features in Figure 7 that obvious differences exist among various data augmentation methods. CT-GAN achieved the optimal overall performance in terms of statistical fidelity. For four mix proportion indicators including cement content, sand ratio, water–binder ratio and fly ash content, the distribution curves of CT-GAN-generated data were highly consistent with original data in terms of shape and peak position. Different from GaussianCopula which caused obvious bimodal offset and TVAE which led to out-of-range samples, CT-GAN effectively maintained the original data range and concentration trend. In terms of mechanical properties (compressive strength and splitting tensile strength), CT-GAN accurately captured the unimodal distribution characteristics of original data, with only minor deviations in the tail density estimation.

By contrast, GaussianCopula tended to generate over-dispersed and bimodal data, failing to retain the concentration characteristics of original samples. TVAE frequently produced samples beyond the value range of original data, accompanied by overall distribution offset. SMOTE and Bootstrap could roughly follow the general trend of original data, but performed poorly in depicting distribution details such as multi-peak characteristics and tail features. In summary, CT-GAN had the highest coincidence degree of kernel density curves with original data, which proves its outstanding capability in learning and reproducing the distribution rules of continuous features, and it can provide high-quality data for subsequent model training.

**Figure 8:**
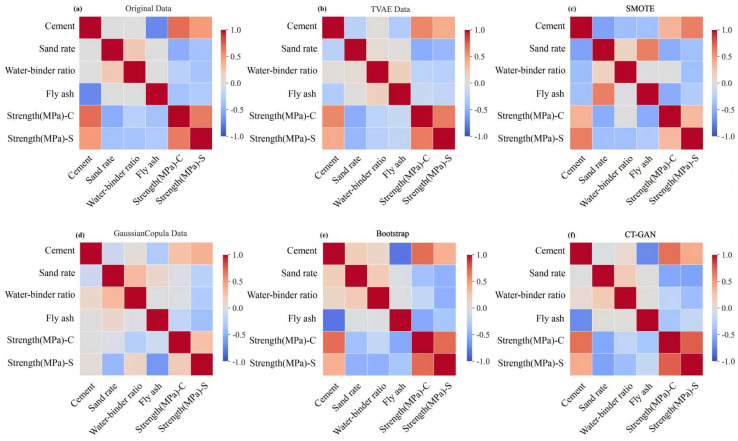
Correlation matrix heatmaps of original and synthetic datasets ((**a**) original data; (**b**) TVAE; (**c**) SMOTE; (**d**) GaussianCopula; (**e**) Bootstrap; (**f**) CTGAN).

To further evaluate the capability of each method in retaining correlations between variables, correlation matrix heatmaps were drawn for original data and synthesized data, as shown in Figure 8. Figure 8 consists of the correlation matrix of original data (a) and those of data generated by TVAE, SMOTE, GaussianCopula, Bootstrap and CT-GAN (b–f). The color gradient from blue to red represents the change from negative correlation to positive correlation, and darker colors indicate stronger correlation.

Overall, the correlation matrix of CT-GAN-generated data had the highest consistency with the original one. It precisely reproduced key correlation patterns of raw data, such as the negative correlation between cement content and strength indicators, as well as the strong positive correlation between compressive strength and splitting tensile strength. Both the sign and magnitude of correlation coefficients were well retained. Other methods showed varying degrees of deviation. TVAE and SMOTE distorted the correlation between partial variable pairs; for instance, the negative correlation between cement and fly ash was obviously weakened, and the negative correlation between water–binder ratio and strength could not be accurately reflected. GaussianCopula over-smoothed the correlations and failed to capture non-linear and high-order relationships among variables. Although Bootstrap retained the general correlation structure, it could not generate new effective information or reproduce complex variable correlations due to simple resampling. The above results demonstrate that CT-GAN excels in learning both marginal distribution and inter-variable dependencies, and can provide statistically representative data for model training.

### 3.4. Decoupling Verification of Sample Quantity and Data Quality

**Figure 9:**
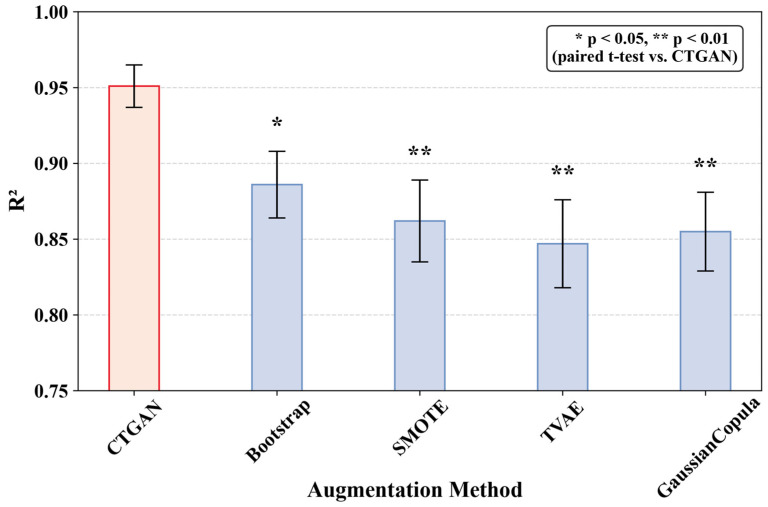
Model performance and statistical test results.

To verify that the performance improvement brought by CT-GAN stems from high-quality data distribution rather than simple expansion of sample quantity, a controlled variable experiment was designed. The original 100 groups of data were fixed as the training set, and 100 groups of data generated by each augmentation method were taken as independent test sets. All test sets had the same sample size, which eliminated the interference of sample quantity and focused on comparing the generalization ability of the model on synthesized data. The XGBoost–LSTM model was adopted and trained five times with different parameter initializations. The mean R^2^, standard deviation, and 95% confidence interval were calculated, and a paired *t*-test was conducted. The experimental results are shown in Table 8 and Figure 9.

Combined with the experimental design and quantitative results, obvious performance gradients were observed among different methods under the condition of unified training set and test set sample size. CT-GAN achieved the highest mean R^2^ of 0.951 with a standard deviation of only 0.014, which was the minimum among all groups. Its 95% confidence interval [0.924, 0.978] was narrow, indicating low data dispersion and excellent stability of model generalization on CT-GAN synthesized data. Bootstrap ranked second with a mean R^2^ of 0.886, a standard deviation of 0.022 and a 95% confidence interval of [0.843, 0.929]. The performance of GaussianCopula, SMOTE and TVAE decreased sequentially, with mean R^2^ values of 0.855, 0.862 and 0.847 respectively. All comparison methods had larger standard deviations and wider confidence intervals than CT-GAN, which proved the poor statistical stability of their generated data and unstable model generalization performance.

The paired *t*-test results further quantified the inter-group differences. The *p*-value between Bootstrap and CT-GAN was 0.016, indicating a significant difference at the *p* < 0.05 level. The *p*-values of SMOTE, TVAE and GaussianCopula against CT-GAN were 0.007, 0.003 and 0.005 respectively, reaching an extremely significant level of *p* < 0.01. Since the sample size of training and test sets was strictly controlled in this experiment, it can be concluded that the performance gain of CT-GAN is attributed to its capability to learn and reproduce the distribution and correlation rules of original data, rather than simply increasing sample quantity. By comparison, Bootstrap, SMOTE, TVAE and GaussianCopula cannot accurately fit the complex statistical characteristics of cemented sand and gravel (CSG) data, thus leading to weaker model generalization.

**Figure 10:**
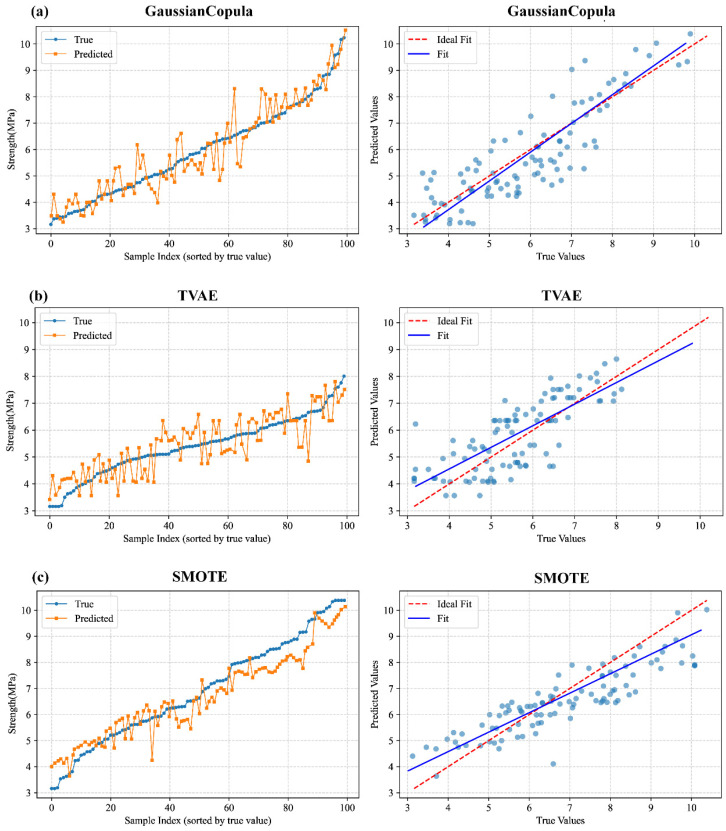

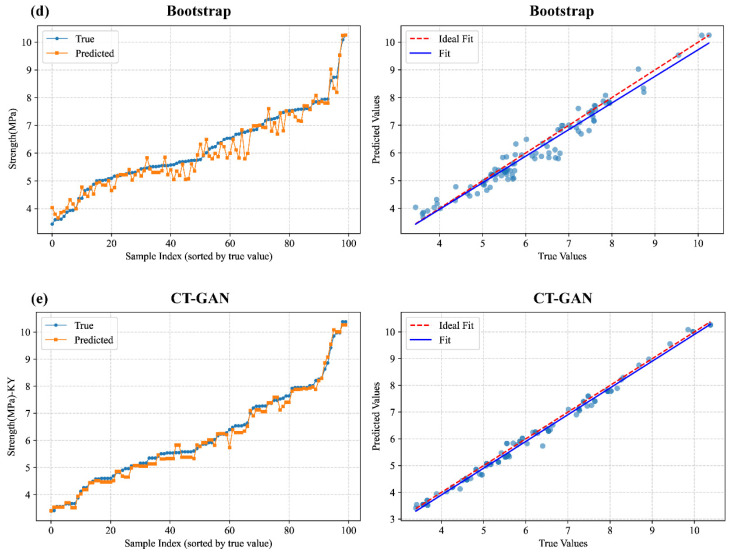
The comparison curve and scatter plot fitting graph of the actual values and the predicted values.

To further visually compare the prediction performance of models on data generated by different methods, the comparison curves of true values and predicted values as well as scatter fitting diagrams were plotted, as shown in Figure 10. The left graphs are error sequences sorted by true values, and the right graphs are scatter plots of true values versus predicted values. The blue solid line represents the model fitting line, and the red dashed line represents the ideal fitting line.

From the overall fitting effect, the prediction results based on CT-GAN data were optimal. In the error sequence graph, the predicted curve was almost completely overlapped with the true value curve with negligible fluctuation, and no systematic deviation existed in low, medium and high strength intervals. In the scatter plot, the fitting line coincided well with the ideal line, and all data points were densely distributed near the diagonal with extremely low dispersion, which reflected high prediction accuracy and consistency. Other methods had different defects. The overall trend of Bootstrap prediction was close to true values, while the error increased in medium and high strength intervals and the scatter dispersion was higher than CT-GAN. GaussianCopula caused frequent fluctuations in prediction sequences, with prominent deviations at low and high values and a large offset of scatter points. TVAE produced systematic prediction bias and generally underestimated medium and high strength values. SMOTE showed segmented errors: overestimation in the medium value range and underestimation in the high value range, and the slope of the fitting line deviated greatly from the ideal line. Although the prediction error of original data was acceptable, the fluctuation of individual samples was larger than that of CT-GAN data under the condition of small samples.

In conclusion, CT-GAN-synthesized data not only maintained consistent statistical distribution and variable correlations with original data, but also improved model prediction accuracy and stability in practical prediction tasks. Its uniform error distribution and strong adaptability to different strength levels fully verify the high quality of data generated by CT-GAN.

### 3.5. Model Performance Analysis Before CSG Data Enhancement

**Figure 11:**
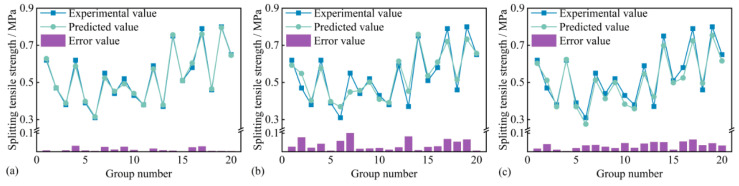
Prediction of compressive strength by each model before augmentation ((**a**) XGBoost–LSTM; (**b**) LSTM; (**c**) XGBoost).

**Figure 12:**
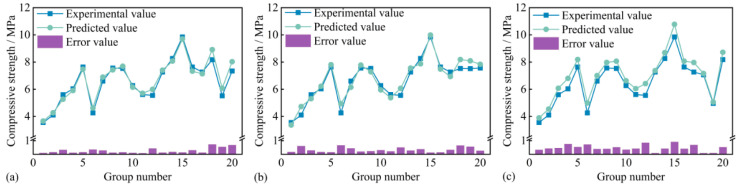
Prediction of splitting tensile strength by each model before augmentation ((**a**) XGBoost–LSTM; (**b**) LSTM; (**c**) XGBoost).

**Figure 13:**
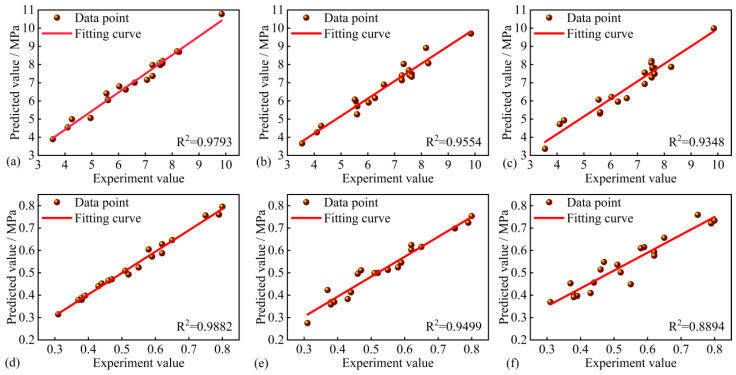
Scatter plot fitting results of strength predictions before data augmentation ((**a**,**d**) XGBoost–LSTM; (**b**,**e**) LSTM; (**c**,**f**) XGBoost for compressive and splitting tensile strengths, respectively).

The combined model XGBoost–LSTM and the base models XGBoost and LSTM were used to predict the compressive strength and splitting tensile strength of the CSG data before augmentation. Figure 11 presents the compressive strength predictions, and Figure 12 presents the splitting tensile strength predictions for each model. Figure 13 shows the scatter plot fitting results on the training and test sets before data augmentation. As shown in Figure 11a and Figure 12a, the XGBoost–LSTM model closely fits the experimental values, with R^2^ of 0.9793 and 0.9882 for compressive and splitting tensile strength, respectively, and absolute errors concentrated within [0, 0.4] and [0, 0.04]. The LSTM model (Figure 11b and Figure 12b) achieves R^2^ of 0.9554 and 0.9499, with errors within [0, 0.6] and [0, 0.06]. The XGBoost model (Figure 11c and Figure 12c) yields R^2^ of 0.9348 and 0.8894, with similar error ranges. These results demonstrate that all three models achieve acceptable prediction accuracy on the original 100-sample dataset, with XGBoost–LSTM exhibiting the best performance.

### 3.6. Model Performance Analysis After CSG Data Enhancement

**Figure 14:**
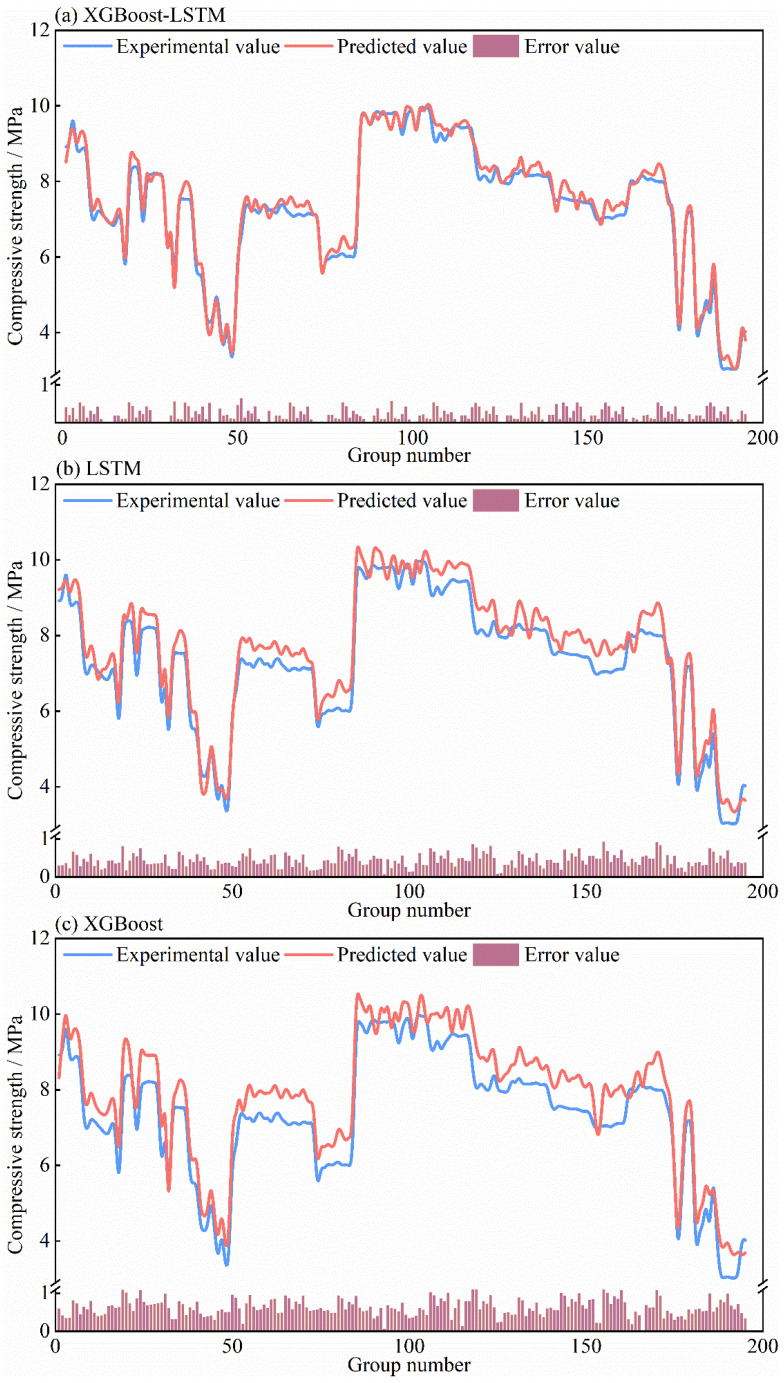
Prediction of compressive strength by each model after augmentation ((**a**) XGBoost–LSTM; (**b**) LSTM; (**c**) XGBoost).

**Figure 15:**
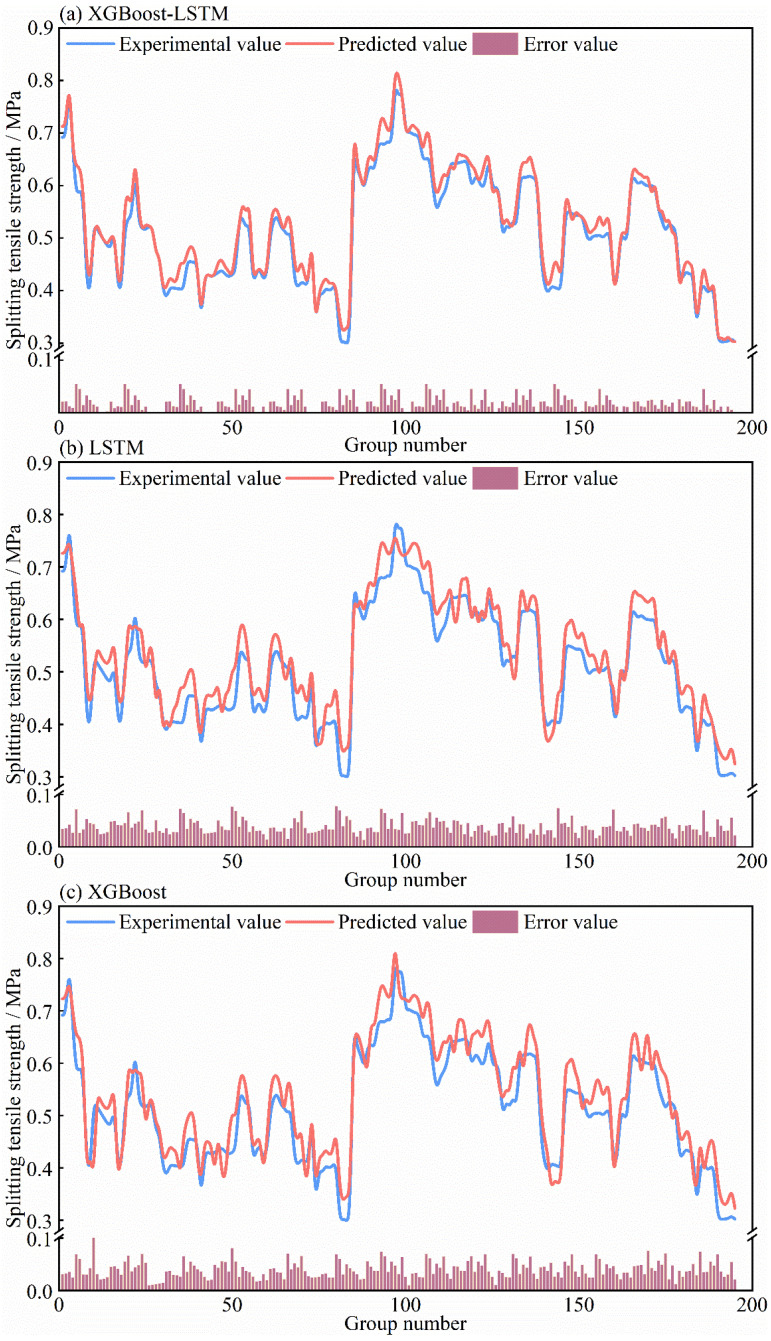
Prediction of splitting tensile strength by each model after augmentation ((**a**) XGBoost–LSTM; (**b**) LSTM; (**c**) XGBoost).

**Figure 16:**
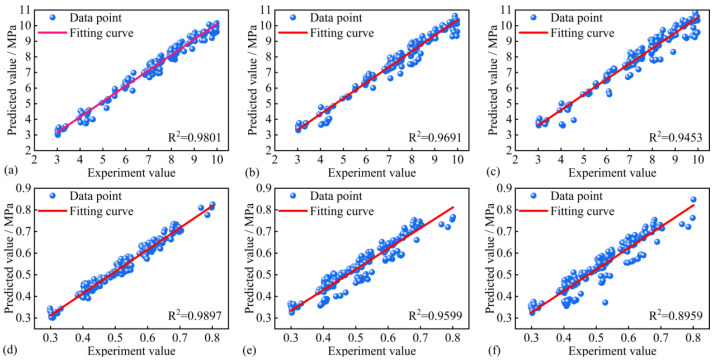
Scatter plot fitting results of strength predictions after CTGAN augmentation ((**a**,**d**) XGBoost–LSTM; (**b**,**e**) LSTM; (**c**,**f**) XGBoost for compressive and splitting tensile strengths, respectively).

Figure 14 presents the compressive strength predictions, and Figure 15 presents the splitting tensile strength predictions for the three models on the CTGAN-augmented CSG data. Figure 16 shows the scatter plot fitting results on the training and test sets after augmentation. As shown in Figure 14a and Figure 15a, the XGBoost–LSTM model closely fits the experimental values, with R^2^ of 0.9897 and 0.9801 for compressive and splitting tensile strength, respectively, representing improvements from 0.9793 and 0.9882 before augmentation. Absolute errors remain concentrated within [0, 0.4] and [0, 0.04]. The LSTM model (Figure 14b and Figure 15b) achieves R^2^ of 0.9599 and 0.9691, with errors within [0, 0.6] and [0, 0.06]. The XGBoost model (Figure 14c and Figure 15c) yields R^2^ of 0.8959 and 0.9453, with similar error ranges. All models exhibit improved prediction accuracy after augmentation compared with the original dataset, confirming the effectiveness of CTGAN data augmentation.

### 3.7. Horizontal Contrastive Analysis

To provide a more comprehensive evaluation of the proposed XGBoost–LSTM model, two additional widely used benchmark models were introduced for horizontal comparison: Random Forest (RF) and Support Vector Regression (SVR). RF is a classical ensemble learning method that has been previously applied to CSG splitting tensile strength prediction, and SVR is an effective regression model for small-sample tasks in civil engineering materials. All models were trained and evaluated on the same augmented dataset under identical experimental settings. The detailed parameter configurations for the additional benchmark models are listed in Table 9.

The MAPE, RMSE, and MAE values of each model are shown in Table 10. The MAPE of all models is below 5.00%, with XGBoost–LSTM exhibiting the lowest MAPE for both compressive strength (4.489%) and splitting tensile strength (4.11%). Moreover, the evaluation metrics for enhanced data remain highly consistent with those for pre-enhancement data, with MAPE differences within 0.1% and RMSE and MAE differences within 0.01, confirming that the CTGAN augmentation preserves the statistical characteristics of the original data across all models.

As shown in Table 11, the XGBoost–LSTM model achieves the highest R^2^ values for both compressive strength (0.9897) and splitting tensile strength (0.9801), along with the lowest RMSE and MAE values among all five models. Specifically, XGBoost alone achieves R^2^ of 0.8959 and 0.9453 for compressive and splitting tensile strength, while RF achieves R^2^ of 0.9205 and 0.9372, and SVR achieves R^2^ of 0.9083 and 0.9245, respectively. Compared with the recurrent model LSTM (R^2^ of 0.9599 and 0.9691), XGBoost–LSTM demonstrates superior performance by leveraging XGBoost’s feature construction capability to enrich the input representation for LSTM. These results indicate that while standalone machine learning models can achieve acceptable prediction accuracy, the proposed XGBoost–LSTM hybrid model provides statistically significant improvements.

The paired comparison results in Table 12 further confirm that the performance differences between XGBoost–LSTM and all four benchmark models are statistically significant (*p* < 0.05 or *p* < 0.01), with all *p*-values well below the 0.05 significance threshold. Among the traditional machine learning models, RF outperforms XGBoost alone, which can be attributed to RF’s bagging strategy that reduces variance through parallel ensemble of independent trees, whereas XGBoost’s boosting strategy may overfit on small datasets. SVR exhibits the lowest performance among all models, likely because the RBF kernel, while effective for capturing local patterns, may not adequately model the global nonlinear relationships across the entire CSG mixture design space. In conclusion, the XGBoost–LSTM combined model exhibits the best predictive performance among all five models, confirming the effectiveness of the two-stage feature construction and refinement architecture for CSG strength prediction.

## 4. Discussion

### 4.1. Advantages

CTGAN has the following advantages for CSG data augmentation: it can generate highly realistic synthetic data, which helps improve the diversity and quantity of data. By introducing a conditional vector, it utilizes specific conditional information from CSG data for data generation. Through adversarial training, the generator and discriminator compete with each other, enhancing the quality of the generated data. The mode-specific normalization mechanism in CTGAN effectively addresses the non-Gaussian and multimodal distributions that are prevalent in CSG data—for instance, the cement dosage in this study exhibits four discrete levels (40, 50, 60, 70 kg/m^3^), while the water–binder ratio and gravel-to-sand ratio each present distinct modal characteristics. Traditional data augmentation methods such as SMOTE generate new samples through linear interpolation in the feature space, which may fail to capture the complex nonlinear relationships among CSG mixture variables. In contrast, CTGAN’s adversarial training framework enables it to learn the joint distribution of all variables simultaneously, preserving both marginal distributions and inter-variable correlations, as demonstrated by the Wasserstein distance and correlation matrix results in Section 3.2.

The XGBoost–LSTM model also presents distinct advantages. The XGBoost stage performs feature construction by learning nonlinear interactions among the four input variables (cement content, sand ratio, water–binder ratio, and fly ash content). This process effectively captures the complex synergistic and antagonistic effects among mixture components that govern CSG strength development. For instance, the interaction between cement content and water–binder ratio determines the effective hydration degree, while the fly ash-to-cement ratio influences pozzolanic reaction kinetics—relationships that are inherently nonlinear and difficult for a single model to fully exploit. By constructing enriched feature representations, XGBoost transforms the raw input space into a higher-level feature space where these interactions are more explicitly encoded. The LSTM stage further processes the XGBoost-constructed features through its gating mechanisms. The forget gate selectively discards irrelevant feature interactions, while the input gate controls the integration of newly constructed information into the cell state. This sequential feature refinement enables the model to identify the most predictive combinations among the constructed features, effectively performing a second level of feature selection.

However, CTGAN also faces some challenges. When dealing with high-dimensional data, training the generator and discriminator may become difficult, requiring more computational resources and time. For certain complex data distributions, CTGAN may require more sophisticated generator and discriminator models to achieve satisfactory generation results. Furthermore, there may be some deviations between the generated synthetic data from CTGAN and the original data, which need to be evaluated and adjusted. In summary, the CSG data augmentation method based on CTGAN, through adversarial training between the generator and discriminator, combined with conditional vectors and Recurrent Neural Network models, can generate realistic CSG data, enhancing both the diversity and quantity of the data.

### 4.2. Limitations

The XGBoost–LSTM model can effectively utilize data from different sources, providing comprehensive information to improve prediction accuracy. It can assess the relative importance of input features, helping identify the most critical factors in CSG performance prediction. However, the model has several limitations that should be critically acknowledged.

First, the model requires large-scale and high-quality datasets, and its performance may degrade if the data quality is poor or the dataset is small. Although the present study demonstrates that CTGAN augmentation can effectively expand a 100-sample dataset to 200 samples while maintaining data quality, the minimum initial sample size required for CTGAN to learn meaningful data distributions has not been systematically investigated. If the original dataset is too small or lacks sufficient diversity in mixture proportions, the CTGAN-generated samples may not adequately represent the true underlying distribution.

Second, a notable limitation of the current study is the relatively narrow range of mixture proportions investigated. The cement dosage ranged from 40 to 70 kg/m^3^, the water–binder ratio from 1.0 to 1.4, and the gravel-to-sand ratio from 0.1 to 0.4. While these ranges cover the typical design space for low-cost CSG dams, the model’s extrapolation capability beyond these ranges remains uncertain. Predictions for mixture compositions outside the training domain should be treated with caution, and future studies should investigate the model’s transferability to broader material compositions and different aggregate sources.

Third, the input features of the current model are limited to four mixture proportion variables (cement content, sand ratio, water–binder ratio, and fly ash content). Other factors that may influence CSG strength, such as aggregate type and gradation, curing temperature and humidity, curing age, and compaction degree, are not included as model inputs. The exclusion of these variables may limit the model’s ability to fully capture the factors governing CSG strength development, and incorporating additional relevant features could potentially improve predictive performance.

Fourth, setting the model hyperparameters requires careful tuning, and the process can be complex. Although this study employed grid search for LSTM hyperparameter optimization, the interaction effects between XGBoost and LSTM parameters were not jointly optimized, which may leave room for further performance improvement.

Fifth, the interpretability of the XGBoost–LSTM model is limited. While the model emphasizes accuracy in predictions, it lacks detailed physical or engineering explanations behind its mechanisms. Although the XGBoost feature importance rankings can provide some insight into which input variables are most influential, the complex interactions within the LSTM layers remain largely opaque. Incorporating interpretability methods such as SHAP (SHapley Additive exPlanations) analysis could provide a deeper understanding of the model’s prediction results and enhance the physical interpretability of the feature interactions.

The model holds potential for improvement. By applying the model to real-time CSG strength prediction, integrating sensor data and real-time monitoring data, it can provide timely warnings and control measures. Improving the model’s predictive performance can be achieved by exploring more relevant features such as humidity, temperature, and pressure, and extracting more effective feature representations. In conclusion, the XGBoost–LSTM combined model has potential value in predicting CSG compressive and splitting tensile strength. By improving data quality, parameter settings, feature engineering, and enhancing the model’s transferability and interpretability in practical application scenarios, its practical applicability and predictive performance can be further enhanced.

### 4.3. Critical Interpretation of Results

#### 4.3.1. Metrics Mechanism Behind CTGAN Augmentation Effectiveness

The improvement in model performance after CTGAN augmentation (R^2^ from 0.9793 to 0.9897 for compressive strength, and from 0.9882 to 0.9897 for splitting tensile strength) warrants a deeper interpretation from a distribution learning perspective. The original dataset of 100 samples, while covering a systematic grid of mixture proportions, likely does not fully represent the continuous underlying strength development function across the input space. CTGAN, through its mode-specific normalization and conditional generation mechanisms, learned the underlying multimodal distribution of CSG data and generated synthetic samples that filled sparse regions of the feature space. This improved the coverage of the training data and reduced the model’s sensitivity to the specific sampling of the original dataset.

The decoupling verification experiment in Section 3.3 provides further insight into this mechanism. When the original 100 samples were fixed as the training set and each method’s 100 synthetic samples were used as independent test sets, CTGAN achieved a mean R^2^ of 0.951 ± 0.014, significantly outperforming Bootstrap (0.886 ± 0.022, *p* = 0.016), SMOTE (0.862 ± 0.027, *p* = 0.007), TVAE (0.847 ± 0.029, *p* = 0.003), and GaussianCopula (0.855 ± 0.026, *p* = 0.005). Since the sample sizes were strictly controlled, this result confirms that the performance gain of CTGAN stems from its superior capability to learn and reproduce the distribution and correlation rules of CSG data, rather than from simple expansion of sample quantity. The narrow standard deviation (0.014) and narrow 95% confidence interval [0.924, 0.978] of CTGAN further indicate that the synthetic data are statistically consistent, providing reliable augmentation for model training.

#### 4.3.2. Physical Context for Model Performance Differences

The relative performance differences among the five prediction models (XGBoost–LSTM > LSTM > RF > XGBoost > SVR, as shown in Table 11) can be interpreted in a physical and methodological context. The superior performance of the recurrent model LSTM over non-recurrent models (XGBoost, RF, SVR) suggests that the sequential processing of feature interactions captures important aspects of CSG strength development that are not fully represented by static feature combinations. However, it should be noted that CSG strength data are not inherently temporal sequences; the sequential processing in LSTM serves as a mechanism for hierarchical feature refinement rather than temporal pattern recognition. This interpretation is consistent with the observation that XGBoost–LSTM outperformed standalone LSTM, confirming that the benefit arises primarily from the two-stage feature construction and refinement architecture rather than from temporal modeling per se.

Among the traditional machine learning models, RF (R^2^ of 0.9205 and 0.9372 for compressive and splitting tensile strength) outperformed XGBoost alone (R^2^ of 0.8959 and 0.9453), which can be attributed to RF’s bagging strategy that reduces variance through parallel ensemble of independent trees, whereas XGBoost’s boosting strategy may overfit on small datasets. SVR exhibited the lowest performance (R^2^ of 0.9083 and 0.9245), likely because the RBF kernel, while effective for capturing local patterns, may not adequately model the global nonlinear relationships across the entire CSG mixture design space.

#### 4.3.3. Implications for Experimental Design

The demonstrated effectiveness of CTGAN augmentation suggests that for future CSG experimental programs, a smaller initial dataset supplemented by CTGAN-generated synthetic samples may be sufficient for developing robust prediction models. However, this potential benefit requires further validation through systematic studies with varying initial dataset sizes (e.g., 30, 50, 80 samples) to establish the minimum sample size threshold for effective CTGAN training. The quality of CTGAN-generated data is fundamentally dependent on the representativeness of the original training data; therefore, the initial experimental design should ensure adequate coverage of the mixture design space, even with a reduced number of specimens.

## 5. Conclusions

In this study, CTGAN was successfully used to enhance CSG data, and the combined XGBoost–LSTM model was employed to predict the compressive and splitting tensile strength of CSG. Compared with the basic models XGBoost and LSTM, the proposed model provides more accurate predictions.

(1) The CTGAN model expands the dataset by learning the features and distribution of the original CSG data, generating synthetic data that are diverse and similar to the original data, while preserving the characteristics and structure of the original data. The quality of the generated data was validated through Wasserstein distance analysis and correlation matrix comparison, demonstrating that CTGAN achieved the highest coincidence degree of kernel density curves with original data among all five augmentation methods (Bootstrap, SMOTE, GaussianCopula, TVAE, and CTGAN) and precisely reproduced key inter-variable correlation patterns. This increases the diversity and coverage of the dataset.

(2) XGBoost–LSTM, XGBoost, and LSTM all demonstrate accurate prediction results for both the compressive and splitting tensile strength of CSG, using both the original and enhanced data. The highest fitting accuracy on the test set reaches 0.9897, 0.9691, and 0.9453, respectively. The decoupling verification experiment confirmed that the performance improvement stems from CTGAN’s high-quality data distribution rather than simple expansion of sample quantity, as CTGAN achieved a mean R^2^ of 0.951 ± 0.014 on independently generated test sets, significantly outperforming Bootstrap (0.886 ± 0.022, *p* = 0.016), SMOTE (0.862 ± 0.027, *p* = 0.007), TVAE (0.847 ± 0.029, *p* = 0.003), and GaussianCopula (0.855 ± 0.026, *p* = 0.005). This indicates the feasibility of machine learning algorithms in predicting the compressive and splitting tensile strength of CSG and demonstrates the effectiveness of the CTGAN model for data augmentation.

(3) The predictive results of the XGBoost–LSTM combined model are closer to the experimental values. Comparing the evaluation metrics, the MAPE, RMSE, and MAE of the XGBoost–LSTM combined model on the validation set are all lower than those of the XGBoost and LSTM models. Compared with two additional benchmark models, RF and SVR, XGBoost–LSTM also achieved superior performance (R^2^ of 0.9897 vs. 0.9205 and 0.9083 for compressive strength, and 0.9801 vs. 0.9372 and 0.9245 for splitting tensile strength). The Wilcoxon signed-rank test confirmed that the performance differences between XGBoost–LSTM and all four benchmark models are statistically significant (*p* < 0.05). Therefore, the predictive results of the XGBoost–LSTM model are more accurate than those of the other compared models.

(4) The CTGAN augmentation improved the R^2^ of the XGBoost–LSTM model from 0.9793 to 0.9801 for compressive strength and from 0.9882 to 0.9897 for splitting tensile strength. The evaluation metrics for enhanced data remained highly consistent with those for pre-enhancement data, with MAPE differences within 0.1% and RMSE and MAE differences within 0.01, validating the fidelity of the CTGAN augmentation process. The proposed CTGAN-based data augmentation combined with the XGBoost–LSTM prediction framework provides a validated and reproducible methodology for CSG strength prediction.

This study contributes to the scientific understanding of data augmentation for heterogeneous composite materials by demonstrating that CTGAN’s mode-specific normalization and conditional generation mechanisms can accurately reproduce the complex statistical characteristics of CSG data, including multimodal distributions and nonlinear inter-variable correlations. The decoupling verification methodology separating data quality from data quantity provides a rigorous evaluation paradigm transferable to other data augmentation studies. The two-stage XGBoost–LSTM learning framework, which performs hierarchical feature construction and refinement, offers a new perspective on modeling complex multi-component material systems where input variables interact through nonlinear synergistic and antagonistic mechanisms. These methodological and scientific contributions extend beyond CSG to other data-scarce domains in experimental materials science and engineering.

## Figures and Tables

**Table 1:** Basic properties of aggregate.

Aggregate Type	Particle Size Range (mm)	Water Content (%)	Water Absorption (%)	Crush Value (%)	Bulk Density (kg·m^−3^)	Apparent Density (kg·m^−3^)
Natural pebbles	5–40	0.43	0.68	2.74	1669.3	2350

**Table 2:** Particle gradation of sand.

Screen holes (mm)	4.75	2.36	1.18	0.60	0.30	0.15	0.075	<0.075
Cumulative residue (%)	6.20	20.48	40.54	62.45	86.42	97.24	98.48	100

**Table 3:** Technical indicators of fly ash.

Ignition Loss/%	Specific Surface Area/(m^2^·kg^−1^)	Density/(g·cm^−3^)	Chemical Composition/%				
			Fe_2_O_3_	CaO	MgO	Al_2_O_3_	SiO_2_
2.48	420	2.42	3.87	2.27	0.81	29.09	53.36

**Table 4:** Physical properties of cement.

Specific Surface Area/ (m^2^·kg^−1^)	Density/ (kg·m^−3^)	Setting Time/ min		Compressive Strength/ MPa		Splitting Tensile Strength/MPa	
		Initial Set	Final Set	3 d	28 d	3 d	28 d
382.00	3035.00	143.00	191.00	30.40	49.60	6.30	8.60

**Table 5:** Mix proportion of CSG.

Cement Content/(kg·m^−3^)	Sand Ratio	Water–Binder Ratio	Fly Ash Content/(kg·m^−3^)
40	0.1	1.0	20
50	0.2	1.2	30
60	0.3	1.4	40
70	0.4	--	50

**Table 6:** Parameter configuration of data augmentation models.

Method	Library	Key Parameters	Parameter Values
CTGAN	SDV (CTGANSynthesizer)	Generator/Discriminator Hidden Layers	2 layers × 256 neurons
		Training Epochs	300
		Batch Size	32
		Learning Rate	2 × 10^−4^
		Optimizer	Adam
TVAE	SDV (TVAESynthesizer)	Hidden Dimension	128
		Training Epochs	300
		Batch Size	32
		Learning Rate	1 × 10^−3^
GaussianCopula	SDV (GaussianCopulaSynthesizer)	Marginal Distribution Estimation	Non-parametric Kernel Density
SMOTE	Self-implemented	Number of Nearest Neighbors (k)	5
Bootstrap	Pandas Sampling 100	Sampling Mode Sampling with Replacement	

**Table 7:** Parameter configuration of prediction models.

Model	Module	Parameters	Parameter Values
XGBoost	XGBRegressor	Number of Estimators	100
		Learning Rate	0.05
		Max Depth	6
		Subsample Ratio	0.8
LSTM	Network Structure	Network Structure	Number of LSTM Units
		Dropout Rate	0.2
		Neurons of Fully Connected Layer	32
		Network Structure	Number of LSTM Units
	Training Configuration	Loss Function	MSE
		Optimizer	Adam
		Training Epochs	100
		Batch Size	16
		Early Stopping Patience	20
		Loss Function	MSE
XGBoost–LSTM	First Stage (XGBoost)	XGBoost Parameters	Consistent with standalone XGBoost
	Second Stage (LSTM)	LSTM Parameters	Consistent with standalone LSTM
	Feature Construction	Input Features	Original Features + XGBoost Predicted Values

**Table 8:** Performance and statistical test results of different data augmentation methods on test sets (Statistical significance was assessed by comparison with CT-GAN. * denotes *p* < 0.05 and ** denotes *p* < 0.01).

Method	Mean R^2^ ± SD	95% Confidence Interval	Significance vs. CT-GAN (*p*-Value)
CT-GAN	0.951 ± 0.014	[0.924, 0.978]	—
Bootstrap	0.886 ± 0.022	[0.843, 0.929]	0.016 *
SMOTE	0.862 ± 0.027	[0.810, 0.914]	0.007 **
TVAE	0.847 ± 0.029	[0.791, 0.903]	0.003 **
GaussianCopula	0.855 ± 0.026	[0.805, 0.905]	0.005 **

**Table 9:** Parameter configuration of additional benchmark models.

Mode	Key Parameters	Parameter Values
RF	Number of Trees	200
	Max Depth	10
	Min Samples Split	5
	Min Samples Leaf	2
SVR	Kernel	RBF
	C (Regularization)	10
	Gamma	0.1
	Epsilon	0.01

**Table 10:** Values of model evaluation indexes.

Data	Model	Compressive Strength			Splitting Tensile Strength		
		MAPE/%	RMSE	MAE	MAPE/%	RMSE	MAE
Pre-enhancement data	XGBoost–LSTM	4.500	0.200	0.159	4.15	0.052	0.040
	LSTM	4.630	0.344	0.287	4.30	0.057	0.043
	XGBoost	4.710	0.408	0.353	4.62	0.060	0.046
Enhanced data	XGBoost–LSTM	4.489	0.201	0.153	4.11	0.049	0.038
	LSTM	4.615	0.329	0.279	4.28	0.053	0.040
	XGBoost	4.697	0.398	0.346	4.60	0.058	0.047

**Table 11:** Comprehensive performance comparison of all models on enhanced data.

Model	Compressive Strength			Splitting Tensile Strength		
	R^2^	RMSE	MAE	R^2^	RMSE	MAE
XGBoost–LSTM	0.9897	0.201	0.159	0.9801	0.049	0.040
LSTM	0.9599	0.344	0.287	0.9691	0.057	0.043
XGBoost	0.8959	0.408	0.353	0.9453	0.060	0.046
RF	0.9205	0.362	0.301	0.9372	0.061	0.048
SVR	0.9083	0.378	0.318	0.9245	0.064	0.051

**Table 12:** Statistical significance test results (*p*-values).

Model Pair	Compressive Strength *p*-Value	Splitting Tensile Strength *p*-Value
XGBoost–LSTM vs. LSTM	0.019 *	0.028 *
XGBoost–LSTM vs. XGBoost	0.005 **	0.007 **
XGBoost–LSTM vs. RF	0.007 **	0.009 **
XGBoost–LSTM vs. SVR	0.004 **	0.005 **

Note: * *p* < 0.05; ** *p* < 0.01.

## Data Availability

The original contributions presented in this study are included in the article. Further inquiries can be directed to the corresponding author.
